# Public health system expenditure on motor vehicle collisions in Brazil: an ecological study

**DOI:** 10.1590/acb402525

**Published:** 2025-03-14

**Authors:** Sofia Wagemaker Viana, Ayla Gerk, Sofia Schmitt Schlindwein, Enzzo Marrazzo, Brenda Feres, Lívia Ribeiro, Madeleine Carroll, David Patrick Mooney, Gabriel Schnitman, Cristina Pires Camargo

**Affiliations:** 1 Kursk State Medical University – Kursk (Kurskaya Oblast) – Russia.; 2 Harvard University – Medical School – Program in Global Surgery and Social Change – Boston (MA) – United States of America.; 3 McGill University – Department of Surgical and Interventional Sciences – Montreal – Canada.; 4 Montreal Children’s Hospital – Harvey E. Beardmore Division of Pediatric Surgery – Montreal – Canada.; 5 Universidade Regional de Blumenau – Faculdade de Medicina – Departamento de Cirurgia – Blumenau (SC) – Brazil.; 6 Pontifícia Universidade Católica de Minas Gerais – Institute of Biological Sciences – Faculty of Medicine – Poços de Caldas (MG) – Brazil.; 7 Universidade Federal do Recôncavo da Bahia – Faculdade de Medicina – Departamento de Cirurgia – Santo Antônio de Jesus (BA), Brazil.; 8 Boston Children’s Hospital – Cirurgia Pediátrica – Boston (MA) – United States of America.; 9 Universidade Federal da Bahia – Faculdade de Medicina – Salvador (BA) – Brazil.; 10 Universidade de São Paulo – School of Medicine – Laboratory of Microsurgery and Plastic Surgery – São Paulo (SP) – Brazil.

**Keywords:** Accidents, Traffic, Costs and Cost Analysis, Global Health, Health Policy, Wounds and Injuries

## Abstract

**Purpose:**

To assess the cost of traffic accidents in Brazil and the impact of age/location.

**Methods:**

All patients admitted to a Brazilian hospital due to traffic accidents from January 2012 to December 2022 and cost of hospital services were obtained from the Department of Information Technology of the Unified Health System. Demographic data were collected in the Brazilian Institute of Geography and Statistics database. Parametric and nonparametric data were analyzed. The Kruskal-Wallis’ test and a post-hoc test were used for data comparison. The ARIMA linear regression method for trend estimation.

**Results:**

In Brazil, 1.6 million individuals were involved in traffic accidents between 2012–2022, resulting in a cumulative hospital expenditure of US$ 38 million. The average hospital admission cost during this time was US$ 239.66, but no correlation was found between the cost per capita and driver population density increase. Hospitalization in the Midwest/South was higher.

**Conclusion:**

The economic impact of traffic accidents on the Brazilian public health system is significant. With a high number of victims admitted annually and evident regional and age-related disparities, there is a clear need for comprehensive and cost-effective healthcare strategies.

## Introduction

Traffic accidents represent the eighth-greatest cause of mortality in the world and the leading cause of death in individuals aged from 5 to 29 years old^1^. It is also a major cause of morbidity and disability, having a significant impact on healthcare systems^1^. Approximately 90% of traffic accident mortality occurs in low- and middle-income countries (LMICs), despite having a lower number of registered vehicles when compared to higher-income countries^1^^,^ 2. The risk of road traffic death is over three times higher in LMICs compared to high-income countries (HICs)^1^. Even though progress in reducing the number of traffic accident mortality in LMICs has been observed in some countries, rates remain high, resulting in direct and indirect socioeconomic impacts.

The economic burden of road crash injuries is estimated at roughly 1% of the gross national product in low-income countries (LICs), 1.5% in LMICs, and 2% in HICs^3^. In 2006, the Latin American burden due to disability and death from traffic accidents was approximately US$ 18.9 billion, while HICs reported US$ 453.3 billion in associated costs^4^. Meanwhile, the cost correlated with traffic accidents in the same year in Brazil was US$ 1.9 billion^4^.

The majority of road safety research has primarily originated from HICs, with only 10% of research being done in the settings of LMICs, leaving a gap in understanding road safety issues specific to LMICs5. Due to notable differences in driving culture, legislation, and traffic law enforcement between HICs and LMICs, it is crucial to intensify research efforts in LMICs. This involves generating local knowledge, creating initiatives tailored to their safety needs, and enhancing practices^5^.

To pursue efficient public policies in LMICs, it is crucial to comprehend the current economic impact of traffic accidents, given their implications for countries’ gross domestic product *per capita* and health expenditure *per capita* 4. Nonetheless, the actual economic impact of traffic accidents in Brazil remains unclear, since the correlation between factors such as age and geographic regions remains unexplored. To our knowledge, this is the first study to analyze the impact of age and geographic regions on Brazil’s financial burden due to traffic accidents. This study aimed to assess the cost of traffic accidents in Brazil and the impact of age and location.

## Methods

This is an ecological study with a time series analysis from January 2012 to December 2022. We collected data from the Department of Information Technology of the Unified Health System (DATASUS) and Brazil’s public healthcare system (SUS) Informatics Department across the 26 states and the Federal District, reporting the public sector rates, representing 60–70% of all hospital admissions. Auxiliary data were collected from the Brazilian Institute of Geography and Statistics (IBGE). We collected data on all patients admitted to a Brazilian hospital due to traffic accidents using the International Classification of Disease’s 10th Revision (ICD-10) codes V01-V99 to limit the input exclusively to car accidents. Only secondary, non-identifiable data was collected for this study.

Our population was divided into three age groups to assess the impact of young drivers and the elderly population: ages below 20, between 20 and 64, and over 65. The yearly cost of hospital services per traffic accident patient was also collected from DATASUS. The population affected was determined by comparing DATASUS morbidity and mortality with age groups and population density at the national and regional levels. We summarized the annual inpatient cost of hospital services for every hospital admission in United States’ dollars using an exchange rate of 0.2 per Brazilian Real (as of November 18th, 2023).

To conceptualize and clarify, Brazil is divided into five regions–North, Northeast, Southeast, South, and Center-West–, each with distinct characteristics and significant regional disparities. The North and Northeast face the most substantial economic and resource challenges, while the South and Southeast are the wealthiest^6^. The Center-West occupies a middle ground in development, experiencing notable growth since the relocation of Brazil’s capital to Brasília^6^. These inequalities have been reflected in many aspects, including health care services in Brazil^7^.

This study describes data according to their nature and distribution: parametric data was represented by mean and standard deviation; nonparametric data was represented by median and interquartile range; and frequency was represented by percentage. We used the Kruskal-Wallis’ test to compare data, followed by a post-hoc test if the data reached significance. To analyze costs, we divided the costs of each region by the population to have a proportional cost range. The statistical test standard α = 0.05 when *p* < 0.05 was considered statistically significant. All the hypothesis tests were two-tailed. To estimate trends, we used the ARIMA linear regression method. This is a method outlined for data that may be influenced by serial autocorrelation, which often occurs in population data measurements. To standardize by population, we plot the regional number of traffic accidents by regional population.

The data collected was registered using the Microsoft Excel software (Microsoft, United States of America). For statistical analysis, this study used STATA v.18 (Stata Statistical Software: Release 18, College Station, TX, United States of America). Additional analyses and graphics were developed using Statistical Package for the Social Sciences (SPSS) 25.0 software (IBM Corp. Released 2017, IBM SPSS Statistics for Windows, Version 25.0, Armonk, NY, United States of America).

## Results

### Total number of traffic accidents

**Figure 1:**
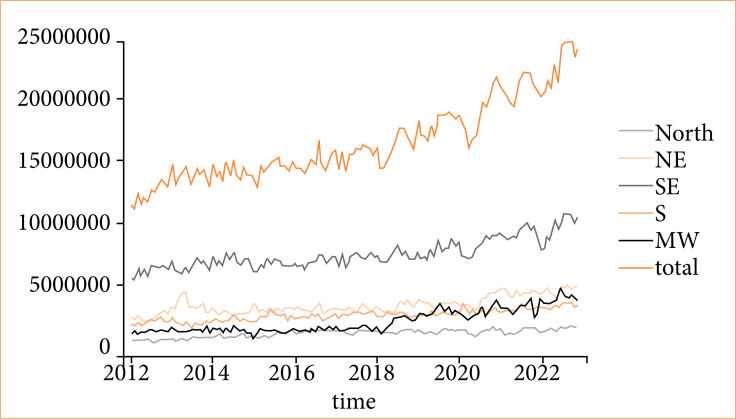
Time series of total traffic accidents by region from 2012 to 2022.

Source: Elaborated by the authors.

The total number of traffic accidents in Brazil has progressively increased in the last 10 years, including the years of the COVID-19 pandemic (Fig. 1). The Southeast region had the highest increase in traffic accidents, doubling in number in 10 years. Meanwhile, the North region displayed only a marginal increase in accidents during the decade.

### Total traffic accident costs

**Figure 2:**
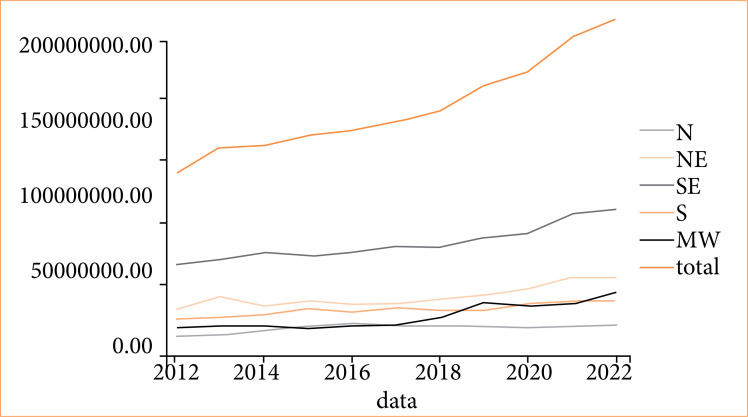
Time series analysis of inpatient costs by region and year.

Source: Elaborated by the authors.

The growth in the number of total accidents caused a direct increase in the total cost of hospitalizations due to traffic accidents in the country. Between 2012 and 2022, the total cost of hospitalizations due to traffic accidents in Brazil was US$ 382,518,146.15, representing a yearly cost of over US$ 38 million in hospital services. With a total number of 1,596,099 people admitted due to traffic accidents, the cost for every hospital admission in the country in the past 10 years was US$ 239.66. This per patient cost rose from US$ 217.02 to US$ 280.98 during the past decade, driven by an increase in all regions and all age groups, except for the population over 60 in the Southern region after 2018. While the overall population increased in all regions, this growth did not reflect a proportional increase in the cost of hospitalizations due to car accidents (Fig. 2).

### Cost of traffic accidents by region and age group

**Figure 3:**
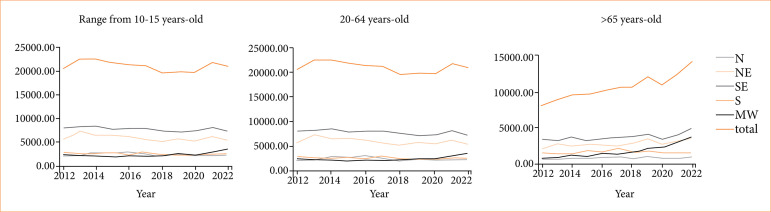
Cost analysis by traffic accident by region and age range (in US dollars).

Source: Elaborated by the authors.

We analyzed the costs of traffic accidents by region and by age group, as shown in Fig. 3. The Southeast is the most populous region of the country, with 42,4% of the country’s population, and it generated the total cost of US$ 197,120,631.32, representing the highest cost per region in all age groups. In the meantime, the Midwest represented the least populated region, with 7.8% of the country’s population and the total cost of US$ 59,521,018.55 due to traffic accidents.

### Proportional costs by region and age group

**Figure 4:**
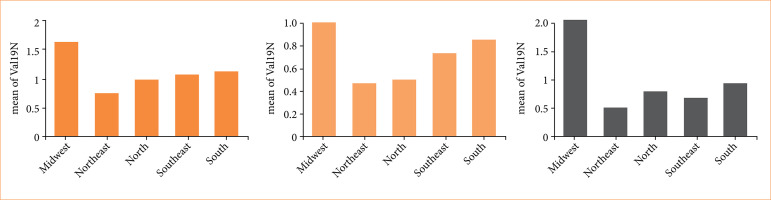
Cost *per capita* due to traffic accidents hospitalizations, by region and age range (in US dollars). **(a)** Under 20 years old age group; **(b)** age group between 20–64 years old; **(c)** over 65 age group.

Source: Elaborated by the authors.

The costs in the Midwest and the South were greater in all age groups than in other regions, while the Northeast presented the lowest costs in all age groups (Fig. 4). For the age group under 20 years old, the Northeast presented the lowest expenditure, with a significant difference when compared to all other regions (*p* < 0.001). We also demonstrated a significant difference between the North’s costs and the Southeast (*p* = 0.03), the South (*p* = 0.001), and the Midwest (*p* = 0.0001). The age group of people between 20–64 years old also presented a significant difference between the costs in the Northeast compared to the South (*p* < 0.001) and the Midwest (*p* = 0.005). The comparison in the older age group showed a significant difference between the Northeast and the North (*p* = 0.04), South (p < 0.001) and Midwest (*p* = 0.001).

For the age group under 20, the Midwest presented the highest cost *per capita*, with a two times higher cost when compared to the North and Northeast (*p* < 0.0001). The South also presented a significant difference in this age group, with a 1.7 times higher expenditure when compared to the lowest cost regions (*p* < 0.001). The Midwest also represented the region with the highest cost *per capita* in the 20–64 years old age group, with a more than two times higher value when compared to the Northeast (*p* = 0.005) and 1.8 higher costs compared to the South (*p* < 0.001). However, the Midwest presented the highest differences in the age group over 65; it had four times the value of expenditure *per capita* compared to the Northeast, where the cost was lowest (*p* = 0.001). The South also had a significant difference from the Northeast, with almost double the cost *per capita* (*p* < 0.001).

## Discussion

The World Health Organization reports approximately 1.35 million deaths per year due to road traffic accidents globally, making it the leading cause of death among individuals aged 5 to 29 years old and representing the eighth cause of mortality among the general population^8^. Over the past decade, Brazil has witnessed a progressive increase in traffic accidents, including during the COVID-19 pandemic, despite ongoing public health efforts to mitigate this issue. Notably, measures such as the Dry Law (Law No. 12.760/2012) enacted in December 2012, which aimed at regulating drunk driving, have been intensively enforced, particularly in the Southeast, where the decline in the death rates due to traffic accidents was most significant^9^^–^ 11. According to Banstola et al.^12^, the most relevant preventive action to avoid traffic accidents is the reduction of alcohol consumption before driving. These measures may ensure a net saving effect of up to US$ 4.14 per disability-adjusted life years (DALYs) in LMICs^12^. The Dry Law and other legislative efforts to reduce traffic accidents, such as speed limit control, appear to have decreased, yet not significantly restricted traffic-related incidents^2^.

The growth in the number of total accidents caused a direct increase in the total cost of hospitalizations due to traffic accidents in the country. Between 2012 and 2022, the total cost of hospitalizations due to traffic accidents in Brazil represented a yearly cost of over US$ 38 million in hospital services, covering approximately 1.6 million individuals. The average hospital admission cost *per capita* was US$ 239.66, distributed unevenly between the different regions and age groups assessed in this study. In the past 10 years, all regions and age groups saw an increase in this *per capita* cost, but the Midwest’s population over 65 after 2018 was a significant contributor. While the overall population size grew in all regions, the growth did not represent a proportional increase in the cost of hospitalizations due to car accidents.

The proportional cost analysis by region and age demonstrated that traffic accident hospitalization expenditures in the Midwest and South were higher than in other regions, even though they are not the most densely populated regions. These disparities were consistent across different age groups, with the Northeast generally incurring the lowest expenditures. Statistical analyses further confirmed significant cost differences between regions, highlighting a complex interplay of demographic factors and healthcare resource allocation^13^. The Southeast region is the most populated in Brazil, with over 84 million inhabitants, representing 42% of the Brazilian population. It is also characterized by its high urbanization and population density^14^.

Cabrera-Arnau et al.^15^ reported on a correlation between accidents and population, in which a higher probability of non-fatal or serious traffic accidents occurred in urban and more densely populated areas due to environmental factors and drivers’ behaviors. Conversely, rural areas surpass urban areas in fatal or serious accidents, owing to the risk behaviors by drivers in rural regions, where there are limited transiting vehicles, a factor that leads to a decrease in fatalities^15^. Interestingly, a study mapping road traffic accidents in the South of Brazil found that in the state with one of the largest national producers of grains, with its predominance of trucks and agricultural vehicles on the highways, which increases difficulty in driving and diminishes road visibility, consequently increasing traffic accidents^16^. These trends, combined with a lack of traffic regulation enforcement in these regions, may explain the results of our study, as the Midwest is considered to be a vastly rural region that presented a progressive increase in the expenditure on traffic accident care, especially when compared to more densely populated regions such as the Northeast and Southeast. This result presented in our study should lead to further analysis, as it appears to show that higher costs of traffic accidents may be more associated with the severity of the injury and less related to the number of accidents.

Other factors outside of population density that may play a role in the expenditure on traffic accidents include the socioeconomic status and demographic characteristics of a region’s population. For example, the rise of the silver economy in the Midwest and South may demand more healthcare costs in any case of hospitalization, since the older population is frequently a victim of injuries with greater pathological consequences due to their physiological fragility and vulnerability^11^. This is worsened by the poor conditions of the sidewalks, the lack of signs, and traffic lights with insufficient time for pedestrian crossing^11^. It has been reported that the burden of disease due to disability is high among the elderly, corroborating our findings that hospitalization costs in the elderly population are proportionally higher when compared to other age groups^11^.

Lastly, local and state public policies may also play a crucial role in preventing traffic accidents. Notably, investment in healthcare in the Midwest and South was lower when compared to the Southeast, while this study observed an increase in expenditures in these regions^17^. Another example was the state governments in the Midwest that implemented discounts for private vehicle acquisition, which may explain the progressive increase in traffic accidents observed in this study^18^^,^ 19.

It is important to note that Brazil is a heterogeneous upper-middle-income country with substantial socioeconomic diversity among its regions. As a continental country, it operates under three health models: the private health system, health coverage, and the universal public health system (SUS), which approximates a universal health coverage framework and provides free health coverage to its population^13^^,^ 20 ^,^ 21. This diversity in healthcare provision and socioeconomic contexts plays a significant role in the regional disparities observed in the impact of traffic accidents.

Our findings emphasize the critical role of a universal healthcare system in managing the economic and social impacts of traffic accidents. However, it also implicates the necessity for comprehensive and cost-effective healthcare strategies to manage the national expenditure related to traffic accidents. Moreover, according to the 2023 Atlas of Violence in Brazil, the most substantial economic burden of traffic accidents falls on the victims themselves, encompassing not only healthcare costs but also the loss of productivity due to injuries or fatalities^22^. This loss of productivity has a cascading effect, impacting both social security systems and the families of the victims, often leading to financial hardship. This, in turn, correlates with the high disease burden in LMIC, accounting for 89% of global DALYs^23^. Future studies are needed to explore out-of-pocket expenditure and the indirect financial burden due to traffic accident victims in Brazil.

Our study has limitations that should be acknowledged. Our database is focused on public healthcare data, potentially underestimating the total impact of traffic accidents in Brazil, where approximately 30% of the population is covered by private healthcare. The reliance on DATASUS as the primary data source also raises concerns about potential underreporting and underrepresentation of certain traffic accident cases. Nonetheless, our study reveals significant regional and age-related disparities in the economic impact of traffic accidents in Brazil. The findings underscore the need for tailored public health interventions and the critical importance of an efficient, universal healthcare system to mitigate the financial and social consequences of traffic accidents across the country.

## Conclusion

There is a significant economic impact of traffic accidents on Brazil’s healthcare system, with the total expenditure of US$ 38 million covering 1.6 million individuals in the last 10 years. Our study revealed a lack of correlation between hospital admission costs and driver population density, pointing to other influencing factors such as socioeconomic status, demographic characteristics, and regional public policies. Our findings also emphasize the critical need for public health intervention and policy reinforcements to mitigate direct and indirect traffic accident impacts, calling for more comprehensive and cost-effective healthcare strategies to address the national expenditure related to traffic accidents.

## Data Availability

Data sharing is not applicable.
